# Blinatumomab vs historical standard therapy of adult relapsed/refractory acute lymphoblastic leukemia

**DOI:** 10.1038/bcj.2016.84

**Published:** 2016-09-23

**Authors:** N Gökbuget, M Kelsh, V Chia, A Advani, R Bassan, H Dombret, M Doubek, A K Fielding, S Giebel, V Haddad, D Hoelzer, C Holland, N Ifrah, A Katz, T Maniar, G Martinelli, M Morgades, S O'Brien, J-M Ribera, J M Rowe, A Stein, M Topp, M Wadleigh, H Kantarjian

**Affiliations:** 1 Department of Medicine, University Hospital, Goethe University, Frankfurt, Germany; 2 Center for Observational Research, Thousand Oaks, CA, Amgen, USA; 3 Department of Hematology/Oncology, Cleveland Clinic, Cleveland, OH, USA; 4 Unità Operativa Complessa di Ematologia, Ospedale dell'Angelo, Mestre-Venezia, Italy; 5 Blood Disease Department (Leukemia Unit), Hôpital Saint-Louis, Paris, France; 6 Department of Internal Medicine - Hematology and Oncology, University Hospital, Brno, Czech Republic; 7 Research Department of Haematology, UCL Cancer Institute, London, UK; 8 Department of Bone Marrow Transplantation and Onco-Hematology, Maria Sklodowska Curie Memorial Cancer Center and Institute of Oncology, Gliwice, Poland; 9 Department of Biostatistics, Uxbridge, Amgen Ltd, UK; 10 Department of Biostatistics, Amgen Inc., Rockville, MD, USA; 11 Blood Diseases Service, Center Hospitalier Universitaire, Angers, France; 12 Department of Clinical Development, Amgen Inc., Thousand Oaks, CA, USA; 13 Institute of Hematology and Medical Oncology “L. and A. Seragnoli” S. Orsola University Hospital, Bologna, Italy; 14 Clinical Hematology Department, ICO-Hospital Germans Trias I Pujol. Jose Carreras Research Institute, Barcelona, Spain; 15 Department of Leukemia, University of Texas MD Cancer Center, Houston, TX, USA; 16 Department of Hematology and Bone Marrow Transplantation, Rambam Medical Center, Haifa, Israel; 17 Department of Hematology and Hematopoietic Cell Transplantation, City of Hope, Duarte, CA, USA; 18 Medizinische Klinik und Poliklinik II, Universitätsklinikums Würzburg, Würzburg, Germany; 19 Department of Medicine, Dana Farber Cancer Institute, Boston, MA, USA

## Abstract

We compared outcomes from a single-arm study of blinatumomab in adult patients with B-precursor Ph-negative relapsed/refractory acute lymphoblastic leukemia (R/R ALL) with a historical data set from Europe and the United States. Estimates of complete remission (CR) and overall survival (OS) were weighted by the frequency distribution of prognostic factors in the blinatumomab trial. Outcomes were also compared between the trial and historical data using propensity score methods. The historical cohort included 694 patients with CR data and 1112 patients with OS data compared with 189 patients with CR and survival data in the blinatumomab trial. The weighted analysis revealed a CR rate of 24% (95% CI: 20–27%) and a median OS of 3.3 months (95% CI: 2.8–3.6) in the historical cohort compared with a CR/CRh rate of 43% (95% CI: 36–50%) and a median OS of 6.1 months (95% CI: 4.2–7.5) in the blinatumomab trial. Propensity score analysis estimated increased odds of CR/CRh (OR=2.68, 95% CI: 1.67–4.31) and improved OS (HR=0.536, 95% CI: 0.394–0.730) with blinatumomab. The analysis demonstrates the application of different study designs and statistical methods to compare novel therapies for R/R ALL with historical data.

## Introduction

Acute lymphoblastic leukemia (ALL) is a rare disease with age-standardized rates ranging from approximately 1 to 2 per 100 000 across various geographies.^1^ Frontline treatments developed for adult patients are often adapted from pediatric approaches and have improved disease prognosis; however, if relapse occurs outcomes are very poor.^2^ In relapsed or refractory (R/R) ALL, complete remission (CR) after salvage treatment was reported in 18–45% of patients and median overall survival (OS) times range from 2 to 8 months.^3, 4, 5, 6, 7^ Reported prognostic factors for worse outcomes among adult R/R ALL patients include older age, later line of salvage treatment, shorter time to relapse from initial achievement of CR, and relapse after receiving allogeneic hematopoietic stem cell transplant (alloHSCT).^3, 5, 7^

Blinatumomab is a BiTE antibody construct that redirects CD3-expressing T-cells to CD19-expressing leukemic cells to induce T-cell activation, proliferation and serial tumor-cell lysis.^8^ Blinatumomab was recently approved by the Food and Drug Administration for patients with R/R ALL largely on the basis of a phase 2 single-arm study (MT103-211) in 189 adult patients.^9, 10^ The study population was comprised of patients with poor prognostic factors, including early first relapse and later lines of salvage therapy, making a direct comparison with published data difficult. After blinatumomab treatment, 43% of patients achieved a response (CR or CR with partial hematological recovery of peripheral blood counts (CRh)) and the median (OS) was 6.1 months.

There are a number of potential approaches for evaluating the relative benefits and risks of a new therapy that uses direct evidence only from single-arm or uncontrolled studies. The strengths and weaknesses of these approaches are summarized in Supplementary Table S1, and include literature review, meta-analysis, evaluation of clinical evidence from a large treating center and pooled analysis of individual patient-level data collected from a number of sites.

Regulatory agencies recognize the option of using external historical controls to demonstrate new treatment efficacy for accelerated approval when a disease is rare, has no satisfactory treatment and the new treatment appears very promising based on preliminary data.^11, 12^ This paper will describe the approach taken to evaluate results of the single-arm clinical trial for the approval of blinatumomab in the USA and Europe.^9, 13, 14^ To provide context for the clinical trial, we conducted a ‘historical comparator' study to evaluate CR and OS with standard of care salvage chemotherapy in adults with Ph-negative, B-precursor R/R ALL.

The historical data set was pooled from European national study groups and large individual sites from Europe and the United States.^13, 14, 15^ Two analytical approaches were used. The first was a weighted analysis, whereby outcomes from the historical data set were weighted according to the frequency distribution of predetermined prognostic baseline factors in the blinatumomab clinical trial population. The second was a propensity score analysis, which created a better balance between historical and blinatumomab-treated patients with respect to important baseline factors, and enabled quantification of differences in outcomes between the two groups. Both methods allowed for more accurate comparisons between historical and clinical trial data than simple descriptive, subgroup or stratified analyses.

## Materials and methods

### Historical comparator study

Full details on the historical comparator study design and data collection are described elsewhere.^13, 14, 15^ The key eligibility criteria were: (1) adult patients with R/R Ph-negative B-precursor ALL, (2) age ⩾15 years at time of initial diagnosis of ALL, (3) initial diagnosis of ALL in the year 1990 or later, (4) no central nervous system involvement at relapse, (5) no isolated extramedullary relapse and (6) no previous treatment with blinatumomab. Anonymized patient data from six national study groups and five large treatment centers (Supplementary Table S2) were forwarded to Amgen for inclusion in the pooled analysis. The anonymized data were checked, pooled and harmonized into a single data set containing predefined variables and outcomes of interest. All authors had full access to the data they provided, and they reviewed and approved summaries of data from their study group or center and the pooled data. Patients had provided informed consent for the collection and use of their data for research purposes in the original study databases. The final protocol was approved by the relevant institutional review boards if applicable, and was registered at clinicaltrials.gov as NCT02003612.

### Blinatumomab clinical trial patient population

Full details of the blinatumomab single-arm phase 2 study (MT103-211) are described elsewhere.^10^ The clinical study population included adult (⩾18 years) patients with Ph-negative, B-precursor R/R ALL (first relapse ⩽12 months of first remission, relapse <12 months after alloHSCT, or no response to or relapse after first salvage therapy or beyond). This study was conducted at sites across Europe and the United States and included 189 adult R/R ALL patients, enrolled over the period 2010–2014. Patients received blinatumomab (9 μg/day for the first 7 days and 28 μg/day thereafter) by continuous intravenous infusion over 4 weeks every 6 weeks (up to five cycles), and were followed for remission, survival and safety outcomes.

### Patient selection from the historical data set

To enable closer comparison with patients from the blinatumomab clinical trial, patients from the pooled historical database were further selected based on key eligibility criteria of the blinatumomab trial: (1) age ⩾18 years at relapse and (2) relapsed within 12 months from initial diagnosis, or relapsed after alloHSCT, or refractory to initial or subsequent treatments, or in second or later relapse. Patients with a first remission duration of >12 months and remaining in first salvage without further relapse were excluded, unless they had a relapse within 12 months of receiving alloHSCT. In addition, patients for whom only palliative care was recorded or had no verifiable record of salvage therapy were excluded. In patients with information on several lines of salvage therapy, only the end points for the latest available salvage therapy were selected for analysis. This mimicked the likely time period when a patient would have entered the blinatumomab study.

### Outcome measures

The primary study outcome for the weighted analysis from the historical data set was achievement of CR after salvage therapy as defined by the individual study groups.^15^ European sites usually defined CR according to standard criteria, that is, bone marrow blasts <5% and no peripheral blast cells or extramedullary manifestations,^7^ and US study sites generally used CR criteria as published for acute myeloid leukemia, involving the complete recovery of peripheral counts.^16^ A secondary outcome was OS, defined as the time from the start of last salvage therapy to death from any cause. The remission end point in the blinatumomab trial was CR (⩽5% bone marrow blasts, platelets >100 000/μl, ANC >1000/μl) and CRh (⩽5% bone marrow blasts, platelets >50 000/μl, ANC >500/μl), without peripheral blasts or extramedullary disease. OS was calculated as time from blinatumomab treatment initiation to death or date of last follow-up.

### Statistical analysis

#### Weighted analysis

Summary estimates of study outcomes from the historical data set were calculated by weighting the frequency distribution of known prognostic factors in R/R ALL, according to standard methods.^17^ Prognostic factors were defined based on published data and the availability of the respective parameters in both data sets, and then used to define patient strata. Six mutually exclusive strata were defined by a combination of age, prior alloHSCT and line of salvage treatment among patients with available CR or OS data.

For each of the six strata, the proportion of patients with a CR was estimated along with an exact 95% confidence interval (CI). The proportions of patients with CR across strata were then pooled into a combined estimate with each stratum weighted to the percentage of patients observed in that stratum from the blinatumomab trial. A 95% CI was estimated for the combined estimate via bootstrapping.^18^

For OS, the Kaplan Meier (KM) median and KM proportions at 6 and 12 months were estimated. The 95% CI for the median within each stratum was estimated.^19^ The 95% CI for the 6- and 12-month KM proportions within each stratum was estimated using the method described in Kalbfleisch and Prentice.^20^ A combined estimate and 95% CI were derived using the stratum-weighted approach described above.

Sensitivity analyses were conducted to see if there were differences in CR and OS over time. Time periods were defined from 2000 onwards, from 2000 to 2004, and from 2005 onwards. Because sites contributed data over varying time periods, and treatment practices at sites may influence response and survival estimates, we also assessed CR and OS by time period only in sites that had data across the entire study period—that is, from 1990 to 2013.

#### Propensity score analysis

A propensity score analysis was used to balance measured characteristics between patients in the blinatumomab clinical trial and patients in the historical data set.^21^ Data from the clinical trial and the historical data set were merged and candidate covariates were selected based on published data regarding their prognostic impact, their ability to discriminate between patients who were and were not treated with blinatumomab, and their availability in both data sets. The available covariates included: (1) age (years), (2) sex (male, female), (3) duration between initial diagnosis and salvage therapy (months), (4) region (USA, Europe), (5) prior HSCT (yes, no), (6) prior number of salvage therapies (1, 2, 3 and 4+ (treated as a continuous variable)), (7) primary refractory and in first salvage (yes, no) and (8) refractory to last salvage therapy (yes, no).

An estimated propensity score (i.e., the predicted probability of participating in the blinatumomab clinical trial if it were being conducted during the period of historical data) was assigned to each patient based on the patient's set of selected covariates.^16, 22^ The balance of covariates between patients in the blinatumomab clinical trial and patients in the historical data set was determined both by regression modeling and by calculation of standardized differences.

In the estimation of treatment effects, propensity scores were used to adjust for differences between patients in the blinatumomab clinical trial and patients in the historical data set using inverse probability of treatment weighting (IPTW) methods.^23, 24^ To address the potential of over-influence of IPT weights among patients with very low probability of participating in the blinatumomab clinical trial, stabilized IPTW (sIPTW) methods were also used as well as trimmed IPTW and sIPTW values whereby outlier values were truncated to maximum non-outlier values.^25^ CR and CR/CRh rates were analyzed using a logistic regression model with a single treatment indicator covariate and propensity score-based weights to adjust for differences between the blinatumomab trial patients and those in the historical data set. The model's coefficient for the treatment effect was used to obtain an odds ratio (OR) and a robust variance estimation (applied using a generalized estimating equation^26^) was used to construct 95% CIs to evaluate the difference in CR and CR/CRh rates between patients in the blinatumomab clinical trial and patients in the historical data set. Similarly, OS was analyzed via a Cox proportional hazards model with a single treatment indicator covariate and using propensity score-based IPTW or sIPTW weights to adjust for differences between the blinatumomab trial patients and patients in the historical data set. A hazard ratio (HR) and 95% CI (using robust variance estimation) were calculated to measure the risk of death among patients in the blinatumomab clinical trial relative to patients in the historical data set.

## Results

### Demographics and characteristics of the historical data set patient population

**Figure 1:**
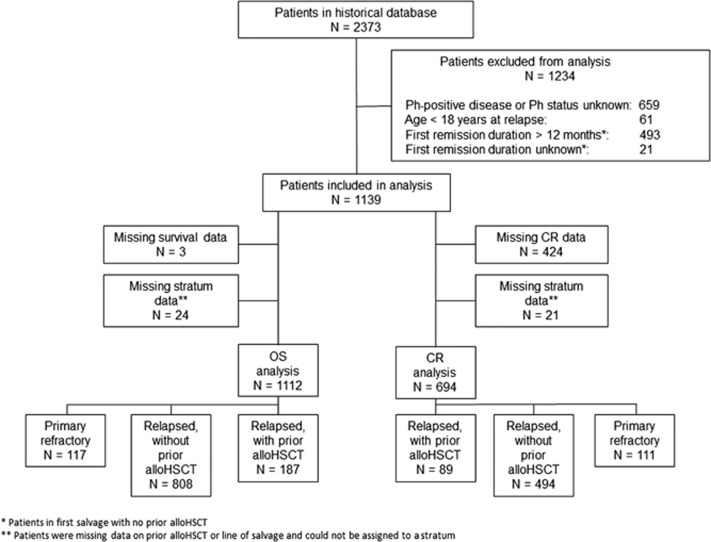
Historical database analysis cohort.

Initially the pooled historical comparator database included 2373 patients with complete remission or survival data (Figure 1). As described above, patients were excluded from the analyses if they did not match the major eligibility criteria of the blinatumomab trial. After further excluding patients with missing outcomes or stratum data (age, treatment history), 694 patients were included in the CR analyses and 1112 in the survival analysis (Figure 1). The number of patients provided by collaborating study groups or sites that met the inclusion criteria ranged from 15 to 233, with 1139 patients providing data for analysis of either CR or OS (Supplementary Table S2).

Demographic characteristics were similar between patients with remission or survival data available in the analysis from the historical data set: a majority were male (approximately 60%); the mean age was 37–39 years, with 45–47% younger than 35 years old (Table 1). The ratio of European to US patients was higher in the OS analysis set than the CR analysis set. This is because two of the European sites provided only OS data. Most patients (64–67%) in both analysis sets were initially diagnosed with ALL in the year 2000 or later. Other characteristics showed only slight differences (Table 1).

The demographic and clinical characteristics of the blinatumomab clinical trial population varied on several characteristics compared with the historical data. There were proportionally fewer patients who were in first salvage, more patients who had a previous HSCT and more patients who had received multiple salvage treatments in the blinatumomab clinical trial (Table 1).

### Weighted analysis

#### Complete remission in the historical comparator patient population

The combined CR rate in the historical data set, weighted to the distribution of patient characteristics in the blinatumomab trial, was 24% (95% CI: 20–27%). In the blinatumomab trial a CR/CRh of 43% (95% CI: 36–50%) was observed. The proportion of patients with CR in the blinatumomab trial was 33% (95% CI: 27–41%).^10^ Stratum-specific rates in the historical data ranged from 17 to 44%, with the lowest rates among patients in second or greater salvage and the highest rates among younger patients in first salvage (Table 2). The weighted CR estimate in the historical data was driven by the low CR in patients in second or greater salvage, who accounted for approximately 50% of the blinatumomab study population. CR rates decreased progressively with each line of salvage therapy (Supplementary Table S3).

Sensitivity analyses were conducted in different time periods (Supplementary Table S4). Compared with the overall population, the CR rate was slightly higher in patients treated from the year 2000 onward (26%, 95% CI: 20–28%), and even higher for patients treated from 2005 onward (30%, 95% CI: 22–37%). However, when limiting analyses to only sites that provided data across the entire time period (i.e., from 1990 to 2013), there was no difference observed in CR rates from 1990 to 1999 (19%, 95% CI: 12–27%) compared with rates from 2000 onward (19%, 95% CI: 12–25%).

#### Overall survival in the historical comparator patient population

The combined median OS in the historical data set, weighted to the distribution of patient characteristics in the blinatumomab trial, was 3.3 months (95% CI 2.8–3.6 months) (Table 3 and Supplementary Figure S1). The weighted 6-, 12- and 36-month survival proportions were 30% (95% CI 27–34%), 15% (95% CI 8–19%) and 6% (95% CI 4–8%), respectively (Table 3). In comparison, the median OS in the blinatumomab clinical trial was 6.1 months (95% CI 4.2–7.5), and the 6- and 12-month survival proportions were 50 and 28%, respectively, with data unavailable for calculation of 36-month survival proportion (Table 3). Stratum-specific median survival ranged from 2.2 to 5.7 months (Table 3). Similar to the CR results, poor survival was observed among patients who were in second or greater salvage and among older patients (Table 3).

Sensitivity analyses for OS by time period showed that OS increased over time, with a median survival of 3.8 months (95% CI: 3.3–4.3) for patients treated from the year 2000 onward and median survival of 4.2 months (95% CI: 3.3–4.9) for patients treated from 2005 onward (Supplementary Table S4). When data were limited to only sites that provided data across the entire time period, median survival increased over time but was not greater than the overall population: median OS was 2.4 months (95% CI: 1.8–2.8) from 1990 to 1999 and 3.2 months (95% CI: 2.7–3.7 months) from 2000 onward.

### Propensity score analysis

#### Covariate balance

The balance in baseline covariates between patients in the blinatumomab clinical study and the historical data was assessed both before and after making adjustments for the propensity score (Supplementary Table S5). Before adjustment, significant differences in six of eight covariates were observed between the two groups of patients. Notably, the blinatumomab patients were more heavily pre-treated than the historical patients (average line of salvage therapy 2.36 vs 1.52, *P*<0.0001) and more were refractory to their last line of salvage (52% vs 23%, *P*<0.0001). Standardized differences were substantially reduced after propensity score adjustment for nearly all of the available covariates (reduction for 7 of 8 covariates). After adjustment there were no significant differences in any covariates between patient groups except for region (more patients from Europe in the historical data set). If important covariates or baseline factors were not adequately balanced, then additional sensitivity analyses were conducted adding those factors as additional covariates into the logistic regression or Cox models. The additional variables were added to the ‘adjusted' models when the *P*-value for the factor was <0.05, or when the standardized difference exceeded 0.10. Generally, balance between the groups was considered to be achieved without the need for further adjustment and the CR and OS outcomes were analyzed.

#### Complete remission

**Figure 2:**
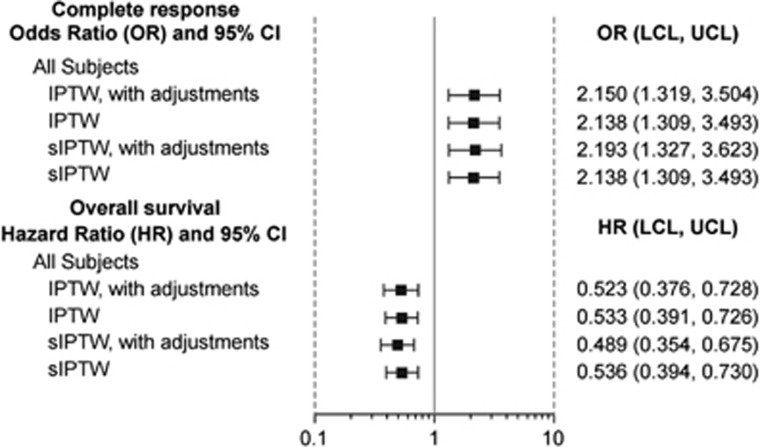
Comparison of complete response and overall survival between blinatumomab clinical trial patients and historical patients. Outcomes were analyzed using both the IPTW and sIPTW approaches: Odds ratio (OR) for achieving a CR/CRh (blinatumomab patients) or CR (historical patients) and hazard ratio (HR) for overall survival.

The proportion of patients from the historical data set achieving CR was compared with the proportion of patients from the blinatumomab trial achieving CR/CRh. The predicted proportions (95% CI) were higher in the blinatumomab patients (49% (33–65%)) than in the historical patients (27% (23–30%)) (Table 4). Figure 2a shows that the odds of achieving a CR were more than doubled with blinatumomab treatment (sIPTW OR=2.68, 95% CI: 1.67–4.31). These findings were also observed across various analyses where subsets of the historical data (patients diagnosed after 2000) were assessed (Supplementary Figure S2).

#### Overall survival

Consistent with the weighted analyses, overall survival was longer among blinatumomab patients (Supplementary Figure S1). Survival proportions were higher in the blinatumomab group than in the historical group after 6 months (58% vs 33%) and 12 months (39% vs 17%) (Table 4). Figure 2b shows that the hazard ratio from the standardized IPTW comparison was 0.54 (95% CI: 0.40–0.73) with an upper bound to the 95% CI below the reference value of 1.0. As with CR, findings for survival were similar in subsets of patients diagnosed after the year 2000 (Supplementary Figure S2).

## Discussion

In special cases when a disease is rare, prognosis is very poor, and there are limited therapeutic options available, single-arm clinical trials may be used as evidence for accelerated drug approvals. Comprehensive evaluation of historical comparator or reference data can provide an additional approach for putting the efficacy of a new therapy into perspective.^11, 12^ In this study, we applied different statistical methods and sensitivity analyses to evaluate the clinical efficacy of blinatumomab against historical data.

Outcomes in previously reported studies of adults with R/R ALL are universally poor, but vary across different populations with different disease characteristics. The overall pooled historical data set (before selection based on blinatumomab study entry criteria) included patients from these studies and showed significant differences in outcomes between different patient subgroups.^15^ For example, higher CR rates were observed in patients with a longer time to first relapse (⩾24 months, 65% vs <6 months, 34%), patients who were younger (15–17 years, 56% vs ⩾65 years, 26%) and patients in first salvage (first salvage, 40% vs third or later salvage, 11%). This is highly relevant for the comparison of different published patient cohorts with variable distribution of these factors. Thus, the direct comparison of major covariates (Supplementary Table S5) demonstrated that almost all of them showed highly significant differences between the historical data set and the blinatumomab trial. These findings emphasize the importance of using appropriate methods to adjust for differences in critical prognostic factors when comparing specific clinical trial results with historical data.

The blinatumomab clinical trial population enrolled patients with particularly advanced disease characteristics, including those with short time to first relapse, prior HSCT and later lines of salvage therapy.^10^ These patients represent a subgroup of an already small patient population.^1^ In order to obtain reliable historical estimates of CR and OS, it was necessary to assemble a large historical data set representative of standard of care in Europe and the USA. From this data set, we were able to select a comparable population of patients to those in the blinatumomab clinical trial.

The weighted analysis and propensity score analysis showed consistently favorable results when comparing blinatumomab to the historical comparator data. CR and median OS in patients treated with blinatumomab were approximately double those in the historical population, both overall and within most of the different patient strata.

Concerns often raised regarding the use of historical comparator data are the influence of potential biases related to selection, misclassification and confounding.^12^ The requirement of rigorous eligibility criteria in the blinatumomab clinical study—such as Eastern Cooperative Oncology Group status of two or lower and absence of abnormal lab values during screening—may increase the chance of better outcomes in the clinical study than the historical data. While it may be possible to use unadjusted historical data when patient populations are sufficiently similar,^27^ the disproportionate number of advanced-stage patients in the blinatumomab trial required methods applied to individual-level data to minimize bias. Selection bias was minimized by use of stringent inclusion criteria into the historical data set and by weighting or adjusting for known prognostic factors. In addition, the historical data set represented adult R/R patients who received standard of care (excluding palliative care patients where possible), without any restrictions to any patient subgroups. Residual confounding may still remain and be difficult to control for, particularly in data sets where differences in important prognostic factors are unknown or not measured in one data set. In this study, nearly all known important prognostic factors were adjusted for in the weighted or propensity score analyses. Missing data on key covariates lead to exclusion of some records from the analyses (Figure 1), which may theoretically bias the overall results. However, our examination of records with missing covariates did not identify significant differences by patient demographic characteristics compared with patients who had complete data (data not shown). Misclassification bias was limited by harmonization of patient-level data in the pooled analysis, which employed common data definitions for disease classification and outcomes characterization.

Although trends in CR and OS may have improved over time,^28^ no new effective treatments emerged for adult R/R ALL over the study period (1990–2013) and the weighting procedures used in this study, which accounted for differences in the distribution of various prognostic factors that varied across calendar period, reduced this effect. Sensitivity analyses showed that when data were restricted to sites that had data across the entire time period, there was no difference in CR between 1990 to 1999 and 2000 onward. Thus, it could be considered that the small differences observed in the weighted analyses by time period may be due to improvements in treatment over time, or that they are simply due to differences in sites contributing data over different time periods.

Even though there is general consistency on how outcomes are reported, heterogeneity with what was labeled as ‘complete remission' by the different study groups and sites is likely present in the historical data. Some groups/sites included only those patients who achieved bone marrow blast reduction below 5% and full recovery of peripheral blood counts when defining CR. Other sites included patients who achieved blast reduction without complete recovery of peripheral blood counts, which represents the standard clinical procedure.^7^ It is therefore very likely that CR rates in the historical comparator group include patients with CRh. This study relied on the customary reporting of CR by each participating group or site for comparison with CR/CRh and CR estimates in the blinatumomab clinical trial data.

The results of the weighted analysis reflect the distribution of patients in the blinatumomab trial, and therefore may not be fully generalizable to studies of other novel therapies in ALL.^29, 30^ Nevertheless, they reveal the poor prognosis of a subgroup of patients with R/R ALL, highlight the value of accumulating historical clinical data for assessing new therapies for this rare, serious illness, and emphasize the need for continued support of multicentre studies, disease registries and collaborative research efforts.

In conclusion, appropriate analytical methods are necessary to address potential biases when comparing historical data with those from clinical trials. By compiling the largest available data set of adult patients with Ph-negative B-precursor R/R ALL, we were able to use two analytical approaches in evaluating the efficacy of blinatumomab vs current treatments. The clinical benefit of blinatumomab in this population will be further evaluated in a confirmatory phase 3 randomized study.^31^

## Figures and Tables

**Table 1:** Demographics and patient characteristics—historical comparator and clinical trial data

	*Historical data set*		*Clinical trial data*
	*Patients with CR data*	*Patients with OS data*	*Blinatumomab trial (MT103-211)*
	N=*694*	N=*1112*	N=*189*^a^
*Sex, n (%)*			
Male	421 (60.7)	644 (57.9)	119 (63%)
Female	273 (39.3)	468 (42.1)	70 (37%)
Mean (s.d.) age, years	38.8 (14.8)	37.4 (14.2)	41.1 (17.3)

*Age group (years)*			
18–34	317 (45.6)	527 (47.4)	90 (48)
35–54	256 (36.9)	428 (38.5)	46 (24)
⩾55	80 (11.5)	115 (10.3)	53 (28)
⩾65	41 (5.9)	42 (3.8)	25 (13)

*Disease status, n (%)*			
Primary refractory			16 (8.5)
In 1st salvage	61 (8.8)	56 (5.0)	4 (2.1)
In 2nd or greater salvage	50 (7.2)	61 (5.5)	12 (6.3)
Relapsed, with prior alloHSCT			64 (33.9)
In 1st salvage	44 (6.3)	130 (11.7)	9 (4.8)
In 2nd or greater salvage	45 (6.5)	57 (5.1)	55 (29.1)
Relapsed, without prior alloHSCT			109 (57.7)
In 1st salvage	245 (35.3)	543 (48.8)	25 (13.2)
In 2nd or greater salvage	249 (35.9)	265 (23.8)	84 (44.4)

*Year of initial diagnosis, n (%)*			
1990 to 1999	246 (35.4)	364 (32.7)	0 (0)
2000 or later	448 (64.6)	748 (67.3)	189 (100)

*Region, n (%)*			
Europe	393 (56.6)	811 (72.9)	95 (50)
USA	301 (43.4)	301 (27.1)	94 (50)

a Numbers by salvage treatment in disease status categories not totalling due to missing data for salvage history (need to verify).

**Table 2:** Stratified and weighted analysis results: comparison of historical data and blinatumomab clinical trial data: CR by strata and weighted to blinatumomab clinical data

*Stratum Definition*		*Blinatumomab trial (MT103-211)*				*Historical data set*			
*Age*	*Disease status*	N	*Stratum proportion (%)*	*Number with CR* a	*CR/CRh % (95% CI)*	N	*Stratum proportion (%)*	*Number with CR*	*CRsg % (95% CI)*
<35	Prior alloHSCT^b^	40	21.2	15	38 (23, 54)	48	6.9	14	29 (17, 44)
<35	In 1st salvage^c^	10	5.3	7	70 (35, 93)	119	17.1	52	44 (35, 53)
<35	In 2nd or greater salvage^c^	40	21.2	17	43 (27, 59)	150	21.6	27	18 (12, 25)
⩾35	Prior alloHSCT^b^	24	12.7	14	58 (37, 78)	41	5.9	11	27 (14, 43)
⩾35	In 1st salvage^c^	19	10.1	5	26 (9, 51)	187	26.9	57	30 (24, 38)
⩾35	In 2nd or greater salvage^c^	56	29.6	23	41 (28, 55)	149	21.5	25	17 (11, 24)
Combined weighted summary		189	100	81	43 (35, 50)	694	100	186	24 (20, 27)

a CR defined as CR+CRh: includes patients with complete remission with full peripheral count recovery (CR, ⩽5% bone marrow blasts, platelets >100 000 cells per μl, absolute neutrophil count >1000 cells per μl) and patients with complete remission and partial recovery (CRh, ⩽5% bone marrow blasts, platelets >50 000 cells per μl, absolute neutrophil count >500 cells per μl).

b All patients with a history of alloHSCT. They could be in 1st, 2nd or greater salvage.

c All patients without a history of alloHSCT.

**Table 3:** Stratified and weighted analysis results: comparison of historical data and blinatumomab clinical trial data: overall survival by strata and weighted to blinatumomab clinical data

*Stratum definition*		*Blinatumomab trial (MT103-211)*						*Historical data set*					
*Age*	*Disease status*	N	*Stratum proportion (%)*	*Median (95% CI) OS in months*	*Survival % (95% CI)*			N	*Stratum proportion (%)*	*Median (95% CI) OS in months*	*Survival % (95% CI)*		
					*6 months*	*12 months*	*36 months*				*6 Months*	*12 Months*	*36 Months*
<35	alloHSCT^a^	40	21.2	7.6 (3.5, 9.4)	59 (41, 73)	28 (11, 47)	—	108	9.7	3.8 (2.9, 4.5)	35 (26, 44)	14 (8, 21)	5 (2, 11)
<35	In 1st salvage^b^	10	5.3	NE (4.1, NE)	80 (41, 95)	53 (17, 80)	—	258	23.2	5.7 (4.9, 6.3)	46 (40, 52)	25 (20, 30)	11 (8, 16)
<35	In 2nd or greater salvage^b^	40	21.2	6.3 (3.7, 12.6)	53 (36, 68)	38 (22, 55)	—	161	14.5	2.9 (2.3, 4.0)	28 (21, 35)	16 (11, 22)	4 (2, 9)
⩾35	alloHSCT^a^	24	12.7	9.3 (3.3, NE)	62 (40, 78)	28 (6, 57)	—	79	7.1	4.0 (2.8, 4.7)	33 (23, 44)	20 (12, 29)	10 (4, 19)
⩾35	In 1st salvage^b^	19	10.1	5.1 (2.8, 7.0)	30 (11, 53)	0.0 (NE, NE)	—	341	30.7	3.7 (3.2, 4.4)	34 (29, 39)	15 (11, 19)	5 (3, 8)
⩾35	In 2nd or greater salvage^b^	56	29.6	3.7 (1.9, 6.5)	39 (26, 51)	19 (8, 32)	—	165	14.8	2.2 (1.7, 2.9)	24 (17, 30)	13 (8, 19)	7 (4, 11)
Combined weighted summary				6.1 (4.2, 7.5)	50 (43, 57)	28 (20, 36)	—	1112		3.3 (2.8, 3.6)	30 (27, 34)	15 (8, 19)	6 (4, 8)

Abbreviation: NE=not estimable

a All patients with a history of alloHSCT. They could be in 1st, 2nd or greater salvage.

b All patients without a history of alloHSCT.

**Table 4:** Propensity score analysis of historical data set and blinatumomab clinical trial data: CR and overall survival^a^

*End point*	*Statistic*	*Historical data set*	*Blinatumomab trial (MT103-211)*
CR/CRh rate	Predicted rate (95% CI)	26.7% (23.4–30.3%)	49.3% (33.4–65.3%)
Overall survival	6-month survival rate (95% CI)	33.4% (31.0–36.1%)	57.6% (54.9–60.4%)
Overall survival	12-month survival rate (95% CI)	17.2% (15.3–19.4%)	39.0% (36.0–42.2%)

a The propensity scores estimates vary slightly compared with weighted analysis and blinatumomab clinical trial due to adjustments made with propensity score modeling. In the weighted analyses, blinatumomab results are not modified and the historical data are weighted to match the distribution of the blinatumomab trial. In the propensity score analyses, both results are modified to match the distribution of a ‘pseudopopulation' in between the blinatumomab and historical control data set.

## Supplementary Material

Supplementary Informations
