# Supplementary material for: Blinatumomab vs historical standard therapy of adult relapsed/refractory acute lymphoblastic leukemia
Source: Blood Cancer J. 2016 Sep 23;6(9):e473–. doi: 10.1038/bcj.2016.84 (PMC5056974; doi:10.1038/bcj.2016.84)

Supplementary Table 1. Different approaches for providing data to be used as comparators for single-arm clinical studies

Method	Strengths	Limitations
**Study-level data**		
Literature review	• Easy and inexpensive<br>• Reviews can be conducted relatively quickly<br>• Studies subject to peer-review process	• Only useful if the published data are reported from patient populations directly comparable with those receiving the new therapy, with outcomes, prognostic variables, and other key variables defined in a similar manner<br>• Standard of care treatments may change over time<br>• Unlikely to be valid in rare diseases
Meta-analysis	• Provides a quantitative summary from published studies<br>• Can be used for direct comparisons	• Comparability depends upon similarities in study populations and definitions of outcomes between studies in the meta-analysis and studies in the new treatment<br>• Heterogeneity across studies in the meta-analysis presents difficulties in how to interpret appropriateness of summarizing outcomes
Single-center experience	• Relatively easy and inexpensive<br>• Data available at patient level and can be harmonized to clinical trial data for new therapy subjects	• Only useful if the single centre data are reported from patient populations directly comparable with those receiving the new therapy, with outcomes, prognostic variables, and other key variables defined in a similar manner<br>• May not reflect general clinical practice
**Patient-level data**		
Pooled analysis – weighted or unweighted	• More precise and robust than study-level analyses<br>• Allows for standardization of variables and application of specific patient selection criteria across different sites<br>• Provides potential for data that are more comparable to the patient population receiving the new therapy	• Data may be collected differently between the historical studies and studies with new therapies, and key study variables may be defined differently<br>• Large differences in study populations may make comparisons difficult<br>• Standard of care treatments may change over time
Matched-pair analysis using a propensity score	• More precise and robust than study-level analyses<br>• Selects for comparable patients between historical and clinical datasets based on predefined variables	• May be difficult to match sufficient numbers of patients, especially if study sizes are small or there are large differences between patient populations

	• Provides potential for data that are more comparable to the patient population receiving the new therapy	• Matching variables may not be clinically relevant<br>• A large amount of data may be lost (from unmatched patients)
Propensity score modelling using IPTW	• Commonly used for comparisons of observational data and previously used in regulatory submissions<br>• Allows more quantitative comparison between the historical data and new clinical studies<br>• Propensity score adjustments create balance between variables common to both datasets	• Requires covariates that are common to both datasets but prognostically important variables may be missing from one dataset<br>• Key study variables may be defined differently<br>• Standard of care treatments may change over time

Supplementary Table 2. Participant data by country

Country	Number of patients eligible for analysis	Number of patients included in weighted analysis	
		All patients (with OS data)	Patients with CR data
Czech Republic	15	14	15
France (3 datasets)	169	169	70
Germany	223	223	170
Italy (2 sites)	63	62	59
Poland	55	49	17
Spain	71	61	62
United Kingdom**	233	233	--
US (3 sites)	310	301	301
**Totals**	1139	1112	694

Supplementary Table 3: CR by line of salvage and weighting to blinatumomab trial (MT103-211)

Line of Salvage*	N	Stratum % in blinatumomab Trial (MT103-211)	Number of patients with CR	Weighted CR % (95% CI)
1st	350	20.1%	119	34 (29, 39)
2nd	197	40.7%	49	25 (19, 31)
3rd	112	22.2%	14	13 (8, 20)
4th or higher	35	16.9%	4	11 (5, 26)
Combined weighted summary**				22 (19, 25)

* Based on the last line of salvage received by each patient. Excludes patients with missing data for prior treatment and prior HSCT.

** Combined weighted summary is the weighted average of salvage lines 1-4, weighted by the proportions in the MT 103-211 study

Supplemental Table 4. Weighted Analysis of CR and OS by Calendar Period in Historical Comparator Data

Study Outcome	Study Sites Included	Time Period	N	Outcome
CR proportion - % (95% CI)	All sites	1990 to2013 (primary analysis)	694	24 (20 – 28)
	All sites with data from 2000 onward	2000 onward	448	26 (21 – 31)
		2000 to2004	263	23 (16 – 29)
		2005 onward	176	30 (22 – 37)
	Limited to sites with data across the whole study period	1990 to1999	244	19 (12 – 27)
		2000 onward	195	19 (12 – 25)
Median OS in months (95% CI)	All sites	1990 to2013 (primary analysis)	1112	3.3 (2.8 – 3.6)
	All sites with data in 2000+	2000 onward	748	3.8 (3.3 – 4.3)
		2000 to 2004	414	3.4 (2.6 – 4.0)
		2005 onward	321	4.2 (3.3 – 4.9)
	Limited to sites with data across the whole study period	1990 to 1999	362	2.4 (1.8 – 2.8)
		2000 onward	440	3.2 (2.7 – 3.7)

Supplemental Table 5. Covariate balance before and after propensity score adjustments, based on survival data analysis set

	Before Adjustments				After Adjustments[1]			
Factor	Historical Dataset (N=1131)	Blinatumomab Trial (MT103-211) (N=189)	Standardized Difference	p-value	Historical Dataset (N=1131)	Blinatumomab Trial (MT103-211) (N=189)	Standardized Difference	p-Value[2]
Age<br>Mean (SD)	37.4 (14.2)	41.1 (17.3)	0.233	0.0014	38.1 (14.5)	36.9 (15.7)	-0.078	0.4694
Female<br>n (%)	477 (42%)	70 (37%)	-0.105	0.1850	475 (42%)	68 (36%)	-0.122	0.2913
Duration since initial diagnosis (months)<br>Mean (SD)	12.2 (12.3)	28.1 (36.5)	0.585	<0.0001	13.8 (15.1)	15.6 (18.0)	0.106	0.1740
Region-- Europe<br>n (%)	822 (73%)	95 (50%)	-0.473	<0.0001	780 (69%)	89 (47%)	-0.452	0.0001
Prior alloHSCT<br>n (%)	209 (18%)	64 (34%)	0.355	<0.0001	238 (21%)	38 (20%)	-0.019	0.8475
Number of prior salvage therapies[3]<br>Mean (SD)	1.52 (0.82)	2.36 (0.99)	0.924	<0.0001	1.64 (0.89)	1.69 (0.87)	0.061	0.5334
Primary refractory and in first salvage<br>n (%)	62 (5%)	4 (2%)	-0.177	0.0587	57 (5%)	19 (10%)	0.194	0.1882
Refractory to preceding salvage<br>n (%)	259 (23%)	98 (52%)	0.627	<0.0001	305 (27%)	51 (27%)	-0.002	0.9833

[1] p-value is from a logistic regression model for the binary variables and a linear regression for the continuous variables
[2] Includes the last line of treatment, which is blinatumomab for blinatumomab subjects

Supplemental Figure 1. Weighted overall survival curves with sIPTW from time of first salvage among adult Ph-negative relapsed/refractory B-precursor ALL patients. Data are shown for a weighted estimate of survival from patients in the historical dataset and for the blinatumomab clinical study patients.

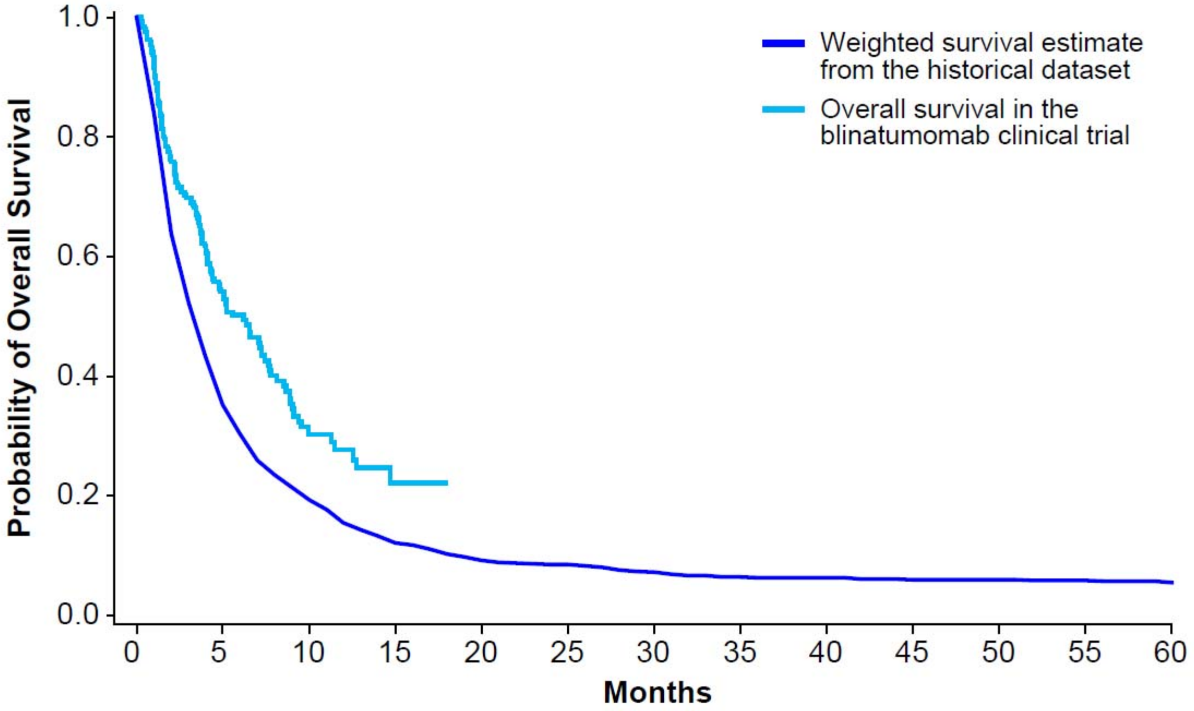

Supplemental Figure 2. Complete response and overall survival between blinatumomab clinical trial patients and historical patients treated from 2000 onward. Outcomes were analysed using both the IPTW and sIPTW approaches: odds ratio (OR) for achieving a CR/CRh (blinatumomab patients) or CR (historical patients) and hazard ratio (HR) for overall survival.

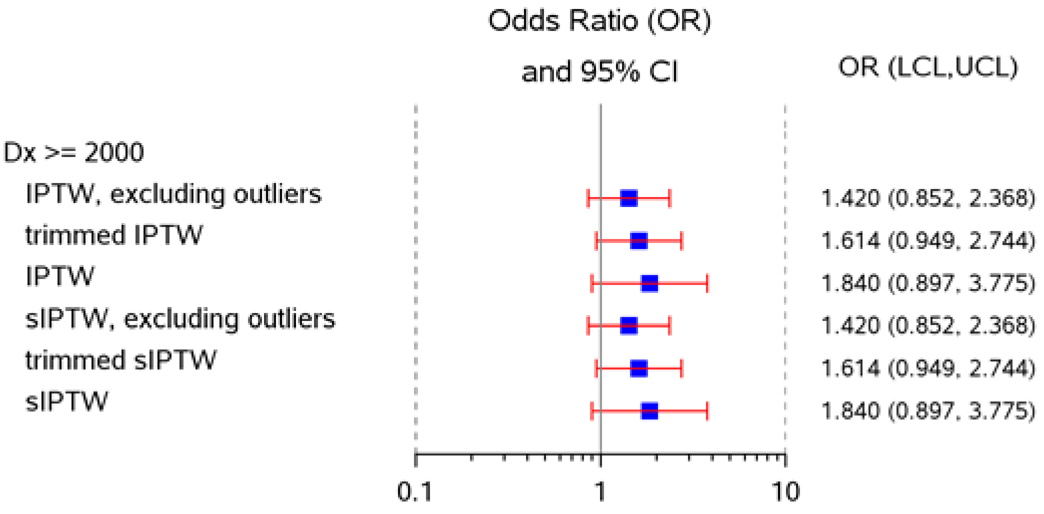

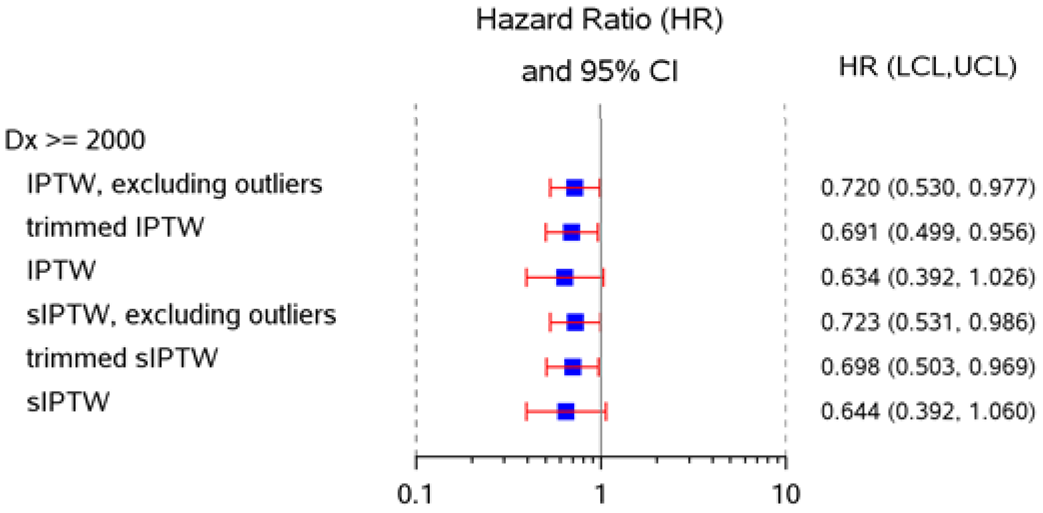

Supplement: Supplementary Informations [file bcj201684x1.pdf]
